# Is the Conditioned Pain Modulation Paradigm Reliable? A Test-Retest Assessment Using the Nociceptive Withdrawal Reflex

**DOI:** 10.1371/journal.pone.0100241

**Published:** 2014-06-20

**Authors:** José A. Biurrun Manresa, Raphael Fritsche, Pascal H. Vuilleumier, Carmen Oehler, Carsten D. Mørch, Lars Arendt-Nielsen, Ole K. Andersen, Michele Curatolo

**Affiliations:** 1 Center for Sensory-Motor Interaction, Department of Health Science and Technology, Aalborg University, Aalborg, Denmark; 2 University Department of Anaesthesiology and Pain Therapy, Inselspital, Bern, Switzerland; 3 Department of Anaesthesiology and Pain Medicine, University of Washington, Seattle, United States of America; Institute of Psychology, Chinese Academy of Sciences, China

## Abstract

The aim of this study was to determine the reliability of the conditioned pain modulation (CPM) paradigm assessed by an objective electrophysiological method, the nociceptive withdrawal reflex (NWR), and psychophysical measures, using hypothetical sample sizes for future studies as analytical goals. Thirty-four healthy volunteers participated in two identical experimental sessions, separated by 1 to 3 weeks. In each session, the cold pressor test (CPT) was used to induce CPM, and the NWR thresholds, electrical pain detection thresholds and pain intensity ratings after suprathreshold electrical stimulation were assessed before and during CPT. CPM was consistently detected by all methods, and the electrophysiological measures did not introduce additional variation to the assessment. In particular, 99% of the trials resulted in higher NWR thresholds during CPT, with an average increase of 3.4 mA (*p*<0.001). Similarly, 96% of the trials resulted in higher electrical pain detection thresholds during CPT, with an average increase of 2.2 mA (*p*<0.001). Pain intensity ratings after suprathreshold electrical stimulation were reduced during CPT in 84% of the trials, displaying an average decrease of 1.5 points in a numeric rating scale (*p*<0.001). Under these experimental conditions, CPM reliability was acceptable for all assessment methods in terms of sample sizes for potential experiments. The presented results are encouraging with regards to the use of the CPM as an assessment tool in experimental and clinical pain.

**Trial Registration:**

Clinical Trials.gov NCT01636440

## Introduction

Under normal conditions, conditioning tonic painful stimulation attenuates the nociceptive response evoked by a test stimulus applied to an extra-segmental body region, a mechanism that was originally described in animals and named descending noxious inhibitory control (DNIC) [1]. It is well known from animal studies that competing inhibitory and facilitatory descending systems are active, which can be assessed individually [2]. In humans, only the net sum between inhibition and facilitation can be measured [3], [4], but since a specific mechanism cannot be discerned, the term conditioned pain modulation (CPM) was suggested to better describe the phenomenon [5]. Recently, a comprehensive review [6] examined several studies displaying evidence that reduced CPM efficiency, reflecting an impairment in pain inhibitory mechanisms, is associated to several chronic idiopathic pain syndromes, e.g. irritable bowel syndrome [7]–[10], temporomandibular disorders [9], fibromyalgia [11]–[15], migraine and tension type headache[16]–[19], as well as to chronic pain states of defined cause, such as chronic pancreatitis [20] and knee osteoarthritis [21].

Despite the vast evidence linking CPM deficiency to several pain syndromes, there is usually no correlation between symptom severity and CPM efficiency [6]. One of the possible explanations for this fact could be related to a large intra- and inter-individual variation of the CPM paradigm itself and/or the methods used to assess it [22]. Before CPM can be used in clinical studies or in drug profiling, it is necessary to determine the reliability of the method, i.e., the amount of measurement error (variation) that is deemed acceptable for the effective practical use of a given measurement tool. To date, a number of studies have attempted to determine the reliability of the CPM paradigm [23]–[30]. However, results from these studies are inconsistent and often contradictory, since several different methodologies have been used to induce CPM, assess its effects and quantify and interpret the underlying test-retest reliability.

Most of the techniques that have been used to assess CPM effects were psychophysical, i.e., verbal reports of pain ratings or thresholds in response to thermal, mechanical and electrical stimulation. As such, the psychophysical techniques are subjective, in the sense that they rely on the subject's self-reported perception, which may introduce an additional source of variation. A viable alternative to assess CPM efficiency is the nociceptive withdrawal reflex (NWR), an electrophysiological measure that has been proven useful in the assessment of spinal nociceptive processing [31], including CPM [3], [4], [32], [33]. One of the main advantages of the NWR is that it is an objective measure, which could potentially result in a more reproducible and stable measure over time. Indeed, test-retest reliability studies of the NWR have revealed good reproducibility over time in healthy volunteers [34] and in chronic low back pain patients [35]. In the light of these reports, the primary aim of this study was to quantify and evaluate the reliability of CPM as assessed with the NWR and other psychophysical pain measures, using hypothetical sample sizes for future experiments as analytical goals. When properly interpreted, the results from this and prior studies are encouraging with regards to the use of the CPM as an assessment tool in experimental and clinical pain.

## Methods

The experiments were performed at the Department of Anesthesiology and Pain Therapy, University Hospital, Inselspital, Bern (Switzerland) between June and August 2012. The study was approved by the ethics committee of the Canton Bern, Switzerland (No. 070/12), registered in the Clinical Trials Protocol Registration System (NCT01636440), and performed in accordance with the Declaration of Helsinki. The protocol for this study and supporting TREND checklist are available as supporting information; see Protocol S1 and Checklist S1.

### Endpoint

The primary endpoint was the reliability of the CPM with the cold pressor test (CPT) as *conditioning* stimulus and electrical stimulation eliciting the NWR threshold as *test* stimulus. Secondary endpoints were the reliability of CPM measures using additional test stimuli: the electrical pain detection threshold and pain intensity ratings to suprathreshold electrical stimulation.

### Design

Since the endpoint of the study was to determine a reference level of the reliability of CPM paradigm, the experiment was designed to minimize the influence of confounding factors (population characteristics, types of conditioning and test stimuli, time between test and retest) in the assessment of measurement error. Thus, CPM was assessed by repeated measures on the same volunteers in two different experimental sessions, with a minimum interval of 1 week and a maximum of 3 weeks between the two measurements, since this is a reasonable time frame for experimental and clinical testing. Moreover, only healthy men were included as volunteers, in order to avoid the possible influence of pain and hormonal changes during menstrual cycle [36], [37]. CPT was chosen as conditioning stimulus because prior studies have shown that it produces better results compared to other techniques, such as mechanical pressure pain or tourniquet pain [23], [24]. The NWR threshold was chosen as primary test stimulus because it is an objective, reliable measure of spinal nociceptive processing [31], [35]. Electrical pain detection thresholds and pain intensity ratings to suprathreshold stimulation were selected as secondary test stimuli because they have also shown good reliability [35] and they can be obtained as a byproduct of the NWR assessment procedure, requiring none or very few additional stimuli.

### Subjects

Volunteers were recruited by advertisement at the Inselspital and the University of Bern. Thirty-nine consecutive pain-free volunteers were tested after obtaining written informed consent. They received 40 Swiss Francs as compensation for their participation in the experiment. Inclusion criteria were: male gender and age of 18–65 years. Exclusion criteria were: presence of any illness, current or past history of drug or alcohol abuse, intake of any psychotropic drug currently or in the last month, chronic alcohol intake, current or regular intake of any drugs that might affect pain or nociception.

### Tests

#### Sample size calculation

The sample size calculation was based on two parameters: the detection of a significant CPM effect and the reliability of that effect over time. The magnitude of the CPM effect was measured as the difference between the NWR threshold during CPT and the NWR threshold before CPT. In a previous study on healthy volunteers, the NWR threshold displayed an average value of 17 mA, a standard deviation of 4 mA, and a range of 5–31 mA [38]. The aim was to detect a minimal difference of 2.0 mA between assessments before and during CPT in both sessions. This resulted in a sample size of 34 subjects, adopting a two-sided significance level of 5% and 80% power. This sample size is also adequate for accurate estimation of reliability measures [39], [40].

#### General methodological aspects

**Figure 1:**
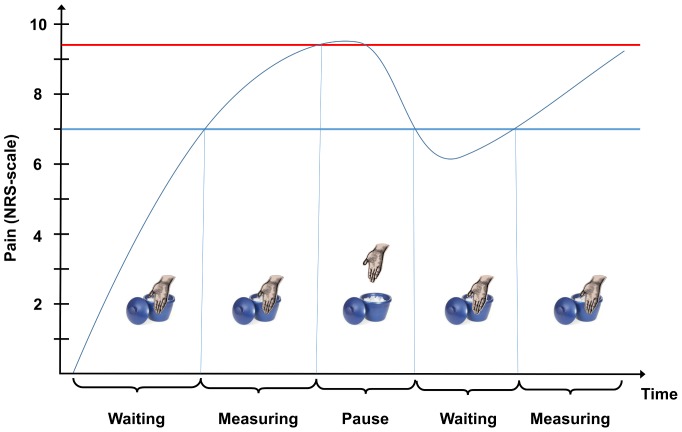
Time course of the experiment. CPT: cold pressor test. NWR: nociceptive withdrawal reflex.

Training sessions of the pain tests were performed before starting the experiment, until the subjects were familiar with the testing procedures. During the experimental sessions, patients were lying in a comfortable supine position, with the upper body elevated by 30°, in a closed and quiet room. The test stimuli were performed on the dominant body side and the conditioning stimulus was performed on the contralateral hand. Volunteers were not allowed to see any read-outs from any instrument. In all volunteers, the second experimental session was performed at the same time of the day (±2 hours in regard to the first experimental session), in order to rule out possible circadian influences on pain sensitivity [41]. All experiments were performed by the same researchers (P.H.V assisted by R.F.) in order to rule out inter-rater variation. The testing sequence is shown in Fig. 1.

#### Electrical stimulation

Electrical stimulation was performed through bipolar surface Ag/AgCl-electrodes placed just distal to the lateral malleolus (innervation area of the sural nerve). Electromyographic (EMG) reflex responses to electrical stimulation were recorded from the middle of the biceps femoris and the rectus femoris muscles (Ag/AgCl-electrodes). Stimulation and EMG recordings were performed using a computer-controlled constant current stimulator (NCS System, Evidence 3102 evo, Neurosoft, Russia). A 25 ms, train-of-five, 1 ms, square-wave pulses (perceived as a single stimulus), was delivered. The current intensity was increased from 1 mA in steps of 1 mA until: 1) a biceps femoris reflex with an amplitude exceeding 20 µV for at least 10 ms in the 60–180 ms post-stimulation interval was detected (NWR threshold); and 2) a pain sensation was evoked (electrical pain detection threshold) [38]. The electrical pain detection threshold was multiplied by 1.5 to obtain the suprathreshold stimulation intensity. Pain intensity ratings after delivery of suprathreshold stimuli were assessed on a numerical rating scale (NRS), where 0 =  no pain and 10 =  worst pain imaginable.

#### Cold pressor test (CPT)

CPT was performed by submerging the volunteer's hand in a container with ice water. The container had an inner compartment and an outer compartment separated by a mesh screen. The mesh screen prevents direct contact between the ice (placed in the outer compartment) and the hand of the subject (placed in the inner compartment). The water was regularly mixed to maintain the temperature in the inner compartment below 2°C, monitored by a thermometer with a digital display (resolution ±0.1°C). The volunteers placed their hands, wide open and up to the wrist, into the inner compartment of the container. They were asked to report when they reach a pain intensity of 7 in the NRS scale. At that point, CPM assessment was performed. If an NRS of 7 was not reached, the assessment was performed after an immersion time of 2 min. The hand was left in the container until all measures were performed or until the pain forced the subject to remove the hand from the container. If the hand was withdrawn from the container during measurements, subjects were asked to re-immerse the hand into the water as fast as possible, and as soon as the pain intensity reached 7 on the NRS scale, the assessment was resumed.

#### Quantification of the CPM effect

The magnitude of the CPM effect, namely ΔCPM, was defined as the difference in NWR threshold during CPT minus NWR threshold before CPT. The electrical pain detection was defined as for NWR threshold. The pain intensity rating to suprathreshold stimulation was assessed as last measure, and ΔCPM was measured as the difference in pain ratings before CPT minus pain ratings during CPT (since pain ratings to the test stimuli are expected to decrease during CPT). Thus, for all measurements, a positive ΔCPM indicates successful modulation and the volunteer is said to respond to CPM testing [22]. Current recommendations also suggest the quantification of CPM as a percent change [5]. However, preliminary analysis showed that data for this variable displayed considerable heteroscedasticity, defined as the situation in which the error increases proportionally to the mean [42]. Heteroscedasticity could not be fully corrected with traditional methods (logarithmic transform, percent difference) and rendered the interpretation of the outcome measure unclear; consequently, the analysis of CPM quantified as percent change was not included in the results.

### Data analysis and statistics

All values are presented as mean ± standard deviation (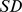). P values smaller than 0.05 were regarded as significant. NWR thresholds, electrical pain detection thresholds and pain intensity ratings to suprathreshold stimulation were compared using repeated measures analysis of variance (RM ANOVA) in SigmaPlot 11 (Systat Software Inc., U.S.A:). Session (first or second), and time (before or during CPT) were regarded as factors.

#### Reliability and sample size estimation

The between-session test-retest reliability was calculated for each test measure before and during CPT (in order to determine if the reliability of the test measures changes during CPT), as well as for the net CPM effect (to determine the actual variation of ΔCPM from session to session). Reliability was assessed using Bland-Altman analysis, coefficient of variation (CV) and intraclass correlation coefficient (ICC) (the respective 95% confidence intervals are reported for CV and ICC). These three methods are the current standard for reliability assessment, and they are reported (alone or in combination) in the vast majority of the reliability studies performed to date [42]–[44]. All these indexes were then calculated in this study for comparison purposes.

Bland-Altman analysis is based on the evaluation of the average vs. the difference of two given measurements, from which the limits of agreement (LoA) can be derived, as the average difference (called *bias*) ±1.96 times the SD of the differences (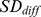). The LoA delimit the range within which 95% of the differences between thresholds/ratings in two sessions may be expected to lie [45], or, in simpler terms, it can be interpreted as the maximum difference that can be expected due to measurement error. Within the context of reliability assessment, the CV represents the within-subject standard deviation 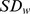 (i.e., the standard deviation of repeated measures over the same subject) expressed as a percentage of the subjects' average threshold/rating [42], [46]. CV is usually reported when the presence of heteroscedasticity is suspected, but it will nevertheless be included in the analysis for comparison with other studies. Finally, the ICC measures the relative homogeneity in thresholds/ratings within sessions in relation to the total observed variation between sessions. In other words, it represents the measurement error relative to the heterogeneity of the subjects [47]. For this analysis, a two-way mixed model using absolute agreement was selected, and the ICC of single measurements was reported. The estimation of sample sizes for potential experiments is a valid alternative to compare the reliability of different methods to assess CPM. Thus, sample sizes for crossover (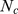)and parallel (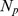) designs were calculated considering standard type I and II error rates of 5 and 20%, respectively, following the guidelines described in [48]. For the crossover design (i.e. the same groups of patients is assessed before and after an intervention), 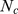 represents the total amount of patients required, whereas for the parallel design case (i.e. patients are divided into treatment and control groups), 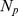
represents the number of subjects required in each group.

#### Relationship between reliability estimates

Even though each reliability estimate measures different aspects of the measurement error, it is natural to think that they are ultimately related in some way. Indeed, all reliability estimates in this study can be linked to the within-subject standard deviation 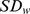 (also called standard error of measurement or typical error), which can be calculated as 
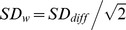
. Then, the LoA can be expressed as 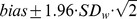, the CV can be expressed as 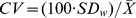 (where 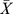 is the average threshold/rating of the sample), and the ICC can be derived as 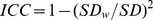. (where 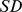 is the standard deviation of the thresholds/ratings of the sample) [44], [45], [49]. Furthermore, sample sizes estimations are also related to reliability estimates, since for a crossover design 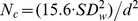, and for a parallel design 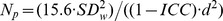 (where 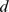 is the desired effect size) [48].

## Results

Thirty nine subjects were recruited for the study. Five subjects were excluded from the study because the NWR could not be elicited. All the remaining 34 subjects completed the study. Their mean age was 27.5±6.8 years and their mean BMI was 23.7±2.5 kg/m^2^. The average interval between sessions was 11.9±1.9 days. All participants reached a NRS of 7 before two minutes. Only in four occasions the hand was withdrawn from the container before the measurements were completed, and in these cases the measurements were resumed shortly thereafter.

### CPM effect analysis

**Figure 2:**
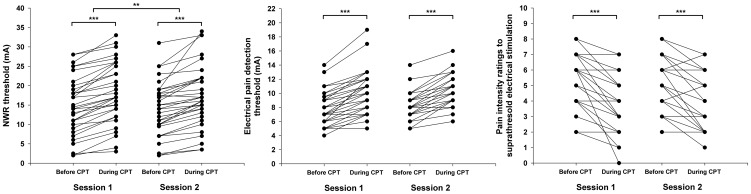
Thresholds before and after the cold pressor test (CPT). Nociceptive withdrawal reflex (NWR) thresholds, electrical pain detection thresholds and pain intensity ratings to suprathreshold stimulation are shown for both sessions. **: *p*<0.01, ***: *p*<0.001.

Results of all performed tests are presented in Table 1. Individual NWR thresholds, electrical pain detection thresholds and pain intensity ratings to suprathreshold stimulation are shown in Fig. 2. For the NWR thresholds, 99% of the measurements during CPT resulted in higher thresholds and 1% resulted in lower thresholds compared to measurements before CPT. The average ΔCPM assessed with the NWR threshold was 3.5±1.9 mA in the first session and 3.5±2.7 mA in the second session. In relation to the electrical pain detection thresholds, 96% of the measurements during CPT resulted in higher thresholds whereas the remaining 4% of the measurements remained unchanged compared to measurements before CPT. The average ΔCPM assessed with the electrical pain detection threshold was 2.3±1.3 mA in the first session and 2.1±1.1 mA in the second session. With regards to pain intensity ratings to suprathreshold stimulation, 84% of the measurements resulted in lower ratings, 13% of measurements remained unchanged and 4% of the measurements resulted in higher ratings during CPT. The average ΔCPM assessed with pain ratings to suprathreshold stimulation was 1.5±1.2 points in the NRS in the first session and 1.4±1.2 points in the second session. Consequently, RM ANOVA revealed a significant CPM effect for all test measures. Specifically, the NWR thresholds and electrical pain detection thresholds were significantly increased (F_(1,33)_  = 108.4, *p*<0.001 and F_(1,33)_  = 195.1, *p*<0.001, respectively), whereas pain intensity ratings to suprathreshold stimulation were significantly decreased (F_(1,33)_  = 68.5, *p*<0.001). Average NWR thresholds were slightly higher in the first session compared to the second session (F_(1,33)_ = 8.0, *p* = 0.008), but the absolute difference (<1 mA) has no practical relevance. No other statistically significant differences between sessions or significant interactions among the factors were detected.

**Table 1:** Variations in the assessment measures due to conditioned pain modulation.

Assessment measure	First session			Second session		
	Before CPT	During CPT	ΔCPM	Before CPT	During CPT	ΔCPM
**NWR threshold (mA)**	14.7±7.2	18.2±7.5	3.5±1.9	13.9±6.8	17.1±7.8	3.3±2.7
**Electrical pain detection threshold (mA)**	7.8±2.2	10.2±2.3	2.3±1.3	8.1±1.9	10.2±2.2	2.1±1.1
**Pain intensity rating to suprathreshold electrical stimulation**	5.3±1.6	3.8±1.7	1.5±1.2	5.3±1.7	3.9±1.7	1.4±1.2

CPT: cold pressor test. ΔCPM: magnitude of the conditioned pain modulation effect. NWR: nociceptive withdrawal reflex. Values are presented as mean ±SD.

### CPM reliability analysis

#### Traditional analysis

**Figure 3:**
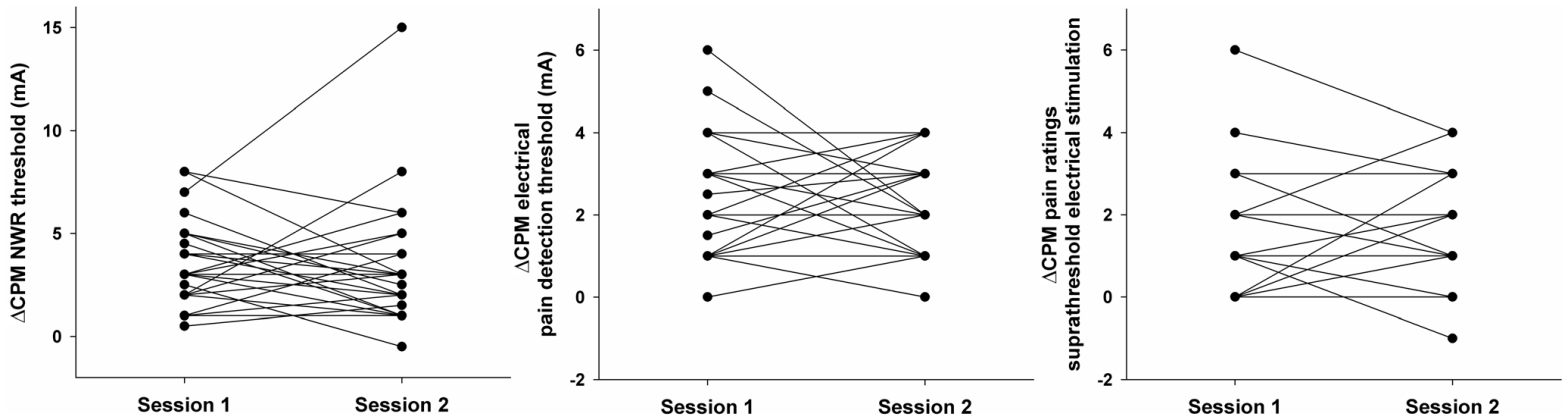
Magnitude of the conditioned pain modulation effect (ΔCPM) for both sessions. Assessment was performed with the nociceptive withdrawal reflex (NWR) thresholds, electrical pain detection thresholds and pain intensity ratings to suprathreshold stimulation.

**Figure 4:**
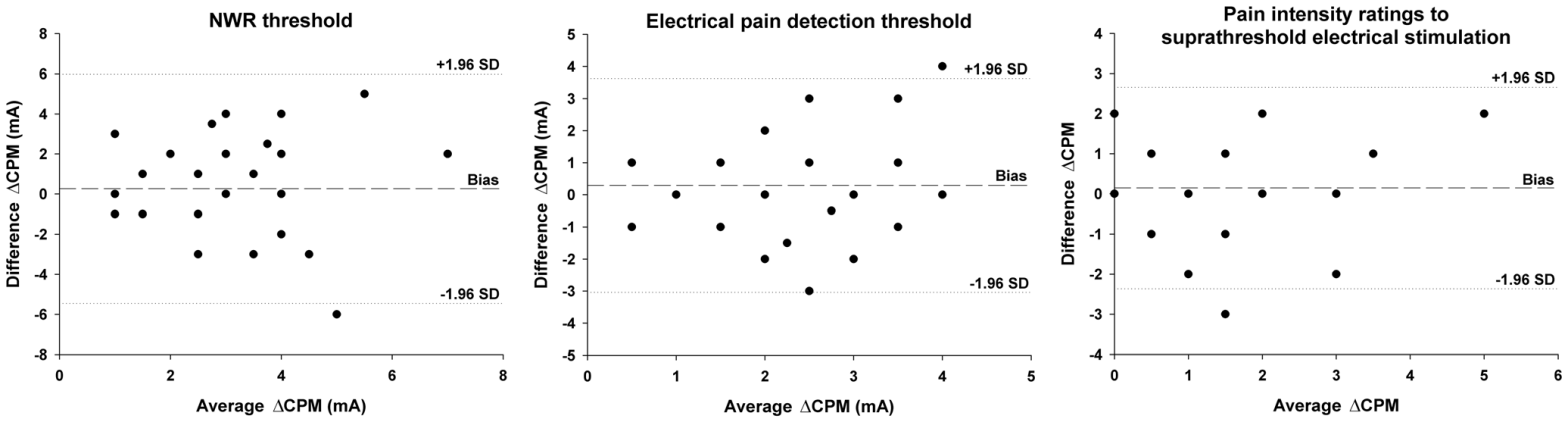
Bland-Altman plots of the magnitude of the conditioned pain modulation effect (ΔCPM). Assessment was performed with the nociceptive withdrawal reflex (NWR) thresholds, electrical pain detection thresholds and pain intensity ratings to suprathreshold stimulation. The dashed line indicates the bias between sessions, whereas the dotted lines indicate the limits of agreement, calculated as ±1.96 times the standard deviation (SD) of the differences in measurements between sessions.

A detailed reliability analysis, including LoA, CV, and ICC values for all performed tests are presented in Table 2. The electrophysiological assessment (the NWR threshold) displayed significantly higher ICC values (i.e. better relative reliability) than the psychophysical measures (electrical pain detection thresholds and pain intensity ratings after suprathreshold electrical stimulation) as demonstrated by the non-overlapping 95% confidence intervals. Moreover, the reliability of the NWR thresholds and electrical pain detection thresholds did not substantially change during CPT, which implies that CPM does not introduce an additional source of variation to these measures; the same cannot be said about pain ratings to suprathreshold electrical stimulation. The reliability analysis of ΔCPM is shown in Table 3. Differences in ΔCPM between sessions are presented in Fig. 3 and Bland-Altman plots are presented in Fig. 4. A visual inspection of Bland-Altman plots did not reveal any apparent signs of heteroscedasticity. Furthermore, no clear statistical differences in any of the reliability indexes could be established for ΔCPM assessed using either electrophysiological or psychophysical measures.

**Table 2:** Reliability of the assessment measures before and during conditioned pain modulation.

Assessment measure	Bland-Altman analysis Bias		CV		ICC	
	(lower LoA – upper LoA)		(95% confidence intervals)		(95% confidence intervals)	
	Before CPT	During CPT	Before CPT	During CPT	Before CPT	During CPT
**NWR threshold (mA)**	0.8	1.1	12.6%	11.5%	0.93	0.94
	(−4.1–5.7)	(−3.6–5.8)	(8.9%–15.4%)	(8.1%–14.2%)	(0.87–0.97)	(0.88–0.97)
**Electrical pain detection threshold (mA)**	−0.4	−0.1	16.9%	14.9%	0.67	0.69
	(−3.8–3.1)	(−4.2–4.0)	(10.1%–21.6%)	(11.3%–17.8%)	(0.43–0.82)	(0.47–0.84)
**Pain intensity ratings to suprathreshold electrical stimulation**	0.0	0.1	14.9%	35.3%	0.85	0.74
	(−1.9–1.9)	(−2.6–2.3)	(8.8%–19.2%)	(8.9%–49.2%)	(0.71–0.92)	(0.54–0.86)

LoA: limits of agreement. CV: coefficient of variation. ICC: intraclass correlation coefficient. CPT: cold pressor test. NWR: nociceptive withdrawal reflex.

**Table 3:** Reliability of the conditioned pain modulation effect.

Assessment measure	Bland-Altman analysis - Bias	CV	ICC
	(lower LoA – upper LoA)	(95% confidence intervals)	(95% confidence intervals)
	ΔCPM	ΔCPM	ΔCPM
**NWR threshold (mA)**	0.3	64.1%	0.26
	(−5.4–6.0)	(39.1%–81.8%)	(0–0.55)
**Electrical pain detection threshold (mA)**	0.3	64.8%	0.09
	(−3.0–3.6)	(48.5%–77.8%)	(0–0.41)
**Pain intensity ratings to suprathreshold electrical stimulation**	0.1	76.2%	0.44
	(−2.5–2.7)	(55.1%–92.7%)	(0.13–0.68)

LoA: limits of agreement. CV: coefficient of variation. ICC: intraclass correlation coefficient. CPT: cold pressor test. NWR: nociceptive withdrawal reflex. ΔCPM: magnitude of the conditioned pain modulation effect.

#### Sample sizes

Given a cohort of pain patients that present impaired pain inhibitory mechanisms, it is reasonable to hypothesize that their average ΔCPM is zero, as shown in prior studies [10], [12], [14], [15]. It would then be relevant to determine how many subjects would be required to demonstrate that a certain drug or treatment is able to return ΔCPM to a given percentage of normal values. Table 4 shows the estimated sample sizes for crossover and parallel experimental designs as a function of the desired effect size 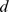, which is estimated as a fraction of the net ΔCPM effect presented in Table 1. For example, it could be considered that an average ΔCPM effect in healthy volunteers would be 3.4 mA if the assessment was performed with the NWR (i.e. there would be 3.4 mA of difference between the NWR thresholds assess before and during CPT). Thus, if it is hypothesized that a new drug or treatment is able to restore up to 50% of normal CPM function in a certain group of patients (i.e. the drug/treatment would improve the average ΔCPM effect from 0 mA to 1.7 mA), then 23 or 62 patients (in a crossover or parallel design, respectively) would be required to successfully verify that hypothesis (with standard type I and II error levels).

**Table 4:** Hypothetical sample sizes of crossover (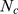) and parallel (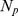) experimental designs as a function of the effect size.

Percentage of return to normal ΔCPM values	NWR threshold		Electrical pain detection threshold		Pain intensity ratings to suprathreshold electrical stimulation	
	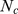	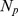	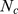	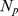	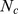	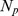
**100%**	6	8	4	5	6	11
**75%**	10	14	8	9	11	19
**50%**	23	31	18	20	24	42
**25%**	93	124	72	79	97	169

CPM: conditioned pain modulation. NWR: nociceptive withdrawal reflex. Note that, despite the actual values obtained, the minimum recommended sample size for a group is 10 [44].

## Discussion

Reliability can be seen as the degree to which a test measures the same way each time it is used under the same condition with the same subjects [50]. Furthermore, reliability can be categorized as relative or absolute [42]–[44], [47]. Relative reliability refers to the degree to which individuals' measurements or scores maintain their position relative to others. Most of the early studies on reliability in medicine only reported relative reliability indexes, such as Pearson's *r*, Spearman's *ρ* and most notably ICC. Absolute reliability refers to the degree to which individuals' measurements or scores vary, assessed across repeated measures. There are several ways to quantify absolute reliability, among which are the within-subject standard deviation, the coefficient of variation, and the 95% LoA proposed by Bland and Altman (and closely related measures, such as the coefficient of repeatability and the minimal detectable change) [42]–[45], [51], [52]. In all cases, reliability studies are useful for estimating the measurement error, which can be employed with several practical purposes, e.g., to determine whether a real change has occurred between measurements, to calculate sample sizes for future experimental/clinical studies or to set criteria for acceptable level of error in measurement tools [40].

A few studies have previously attempted to analyse the reliability of the CPM paradigm, but the results were very inconsistent [23]–[30]. To begin with, there were considerable methodological differences between studies, e.g. population characteristics (gender, age, presence/absence of pain), types of conditioning stimuli (CPT, mechanical or heat pain) and test stimuli (thermal, pressure or electrical thresholds, pain ratings to suprathreshold stimulation), and time between test and retest (from a few minutes to several weeks). Some studies reported good to excellent reliability [25], [28], whereas others informed poor reliability [27], [30], and the rest reported largely mixed results depending on factors such as gender [26], [29], methodology for induction and/or assessment of CPM [23], [24], [26] and time between test and retest [23], [26], among others. In this context, it was not really clear whether the CPM paradigm is reliable or not, or which are the sources of these discrepancies.

However, some of the inconsistencies in these results may be related not only to methodological issues, but also to how reliability is reported and interpreted [53]. First of all, none of the previously mentioned studies checked for heteroscedasticity, which is an essential requirement before further analysis can be carried out [42], [44], [45]. Furthermore, it is recommended that a combination of reliability indexes are analysed to obtain a more comprehensive analysis, since no single estimate is universally appropriate to determine the reliability of a measurement [42], [43]. However, many studies assessing the reliability of CPM only presented ICC and consequently, only relative reliability (e.g. [23], [27], [28], [30]). The reported values in these studies covered practically the whole range of variation for ICC (from 0 to 1), so a clear conclusion cannot be drawn. Often, the reliability of the CPM paradigm was judged based on a comparison with a fixed ICC threshold, and in some of these cases, a positive evaluation was concluded, disregarding the fact that the 95% confidence intervals for ICC were considerably wide (e.g. [23], [29], [30]). Moreover, significance testing of reliability estimates may be misleading if not interpreted properly; for example, given a sufficiently large sample size, an ICC value can be nearly zero but significantly different from zero, or the bias between measurements might be very small yet statistically significant. In both cases, what matters is not just the statistical significance, but also the magnitude of the index [39], [40].

The acceptable level of reliability for each measurement tool depends solely on the actual experimental conditions in order to determine the amount of measurement error that is acceptable for practical use [39], [42], [43]. Thus, universal cut-off thresholds cannot be applied in every circumstance to determine if an assessment method is reliable or not. Nevertheless, the vast majority of studies assessing the reliability of CPM have used fixed thresholds as a reference to establish whether a measure is reliable or not (e.g. [23], [25]–[30]), despite the fact that it is widely ill-advised [42]–[44]. These thresholds are often arbitrary and present a large variation: for ICC they range from 0.6 to 0.9 [27], [28], [54], whereas for CV they range from 10 to 25% [35], [42], [52], [55]. Other factors should also be considered in the interpretation of reliability indexes; for example, ICC values depend on sample size and heterogeneity and on the range of measurement/scores [40], [42]. Large ICC values can mask poor session-to-session consistency when between-subjects variability is high, and a low ICC can be found even when session-to-session variability is low if the sample is very homogeneous [44], [47]. Moreover, even if it is a dimensionless statistic, it is not correct to compare the reliability of two measures using ICC alone: the measure with the largest variation could have a higher ICC if its reliability was determined with a more heterogeneous sample [52]. CV, on the other hand, is sensitive to a shift in scale, and as a consequence, the same test can be shown to be more reliable simply by adding a constant to all scores, as the case of heat/cold thresholds measured in degrees Celsius or Fahrenheit [52]. Finally, it is also relevant to consider the way in which the CPM effect is quantified (either as absolute differences, percent ratio or percent change), since the same CPM test could be deemed reliable or unreliable depending on the quantification of the outcome measure (e.g. [26]).

In summary, many of the inconsistencies in the conclusions of prior studies with regards to the reliability of the CPM paradigm could be partially explained by an inadequate use of the methods to assess reliability and/or an erroneous interpretation of the results. Most of the issues in the interpretation of CPM reliability arise due to the fact that ΔCPM has very particular features as a quantification variable: since it is derived from the difference between two quantities, its range is restricted compared to those of the original assessment methods (as can be noted by comparing the measurement ranges in Figs. 2 and 3). In most cases, this will likely imply two things: ICC values will tend to be comparably low in relation to the original measures, since the within-subject variation will likely be on the same level as the between-subject variation (because of the restricted range of values of ΔCPM). Moreover, CV values will be comparably high: since the offset (i.e., the minimum value that a measurement can take) is eliminated when the differences are computed, the mean ΔCPM (the denominator on the CV ratio) will be closer to zero compared to the original assessment method.

With this in mind, a few concrete examples are presented next in order to clarify the interpretation of the results. From Table 1, the average ΔCPM between the two sessions was 3.4 mA; therefore it would be likely that a subsequent volunteer to be tested shows a ΔCPM magnitude of 4 mA in the first session. If the same volunteer is tested again within 1–3 weeks, then the CV indicates that the *typical* variation for the retest would be within 64% of this value, i.e., in the range of 1.5–6.5 mA (for thorough description of what *typical* means in this context, please refer to [56]). It has to be noted that 64% might seem a relatively high value compared to usual cut off thresholds of 10–20%, but it is a percentage applied on a comparatively small value (since ΔCPM is a differential measure). Furthermore, the LoA indicate that there is a 95% probability that the retest ΔCPM will be in the range of −1.4 to 10 mA, and that any difference that exceeds this range is likely not caused solely by measurement error (note that there is a small chance that CPM will not be elicited at all, as suggested by the negative values). Finally, the ICC value of 0.26 suggests that it is hard to predict whether ΔCPM for a given subject in the retest will be larger or smaller compared to other subjects' ΔCPM (i.e., his relative ranking compared to other subjects is likely to change, as can be seen in Fig. 3). In other words, the within-subject variation is large *relative* to the between-subject variation, a conclusion that should not be used to infer how large the absolute within-subject variation (i.e. the measurement error) really is.

It can readily be seen that the interpretation of reliability measures is not trivial, and it requires a degree of insight into the statistical properties of the variables measured. Furthermore, the decision whether a method is reliable or not for a given purpose should not be made simply by comparison to a predefined, fixed threshold. One the contrary, it depends on the practical experimental conditions of each study and its intended goals (the same method could be sufficiently reliable for some applications and unreliable for others) [43]. However, none of the previously mentioned studies attempted to evaluate the reliability of the CPM paradigm based on clinical or experimental analytical goals. For example, an important use of reliability is to estimate sample sizes for experimental studies [42]–[44], so it follows that a valid criterion to establish whether the reliability of ΔCPM is acceptable or not is by calculating sample sizes for potential experiments [57]. Indeed, it can be seen that the formulas for sample size estimation combine absolute (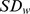) and relative (*ICC*) reliability indexes. It follows that different hypotheses (leading to different effect sizes) will render different results [28], [58]; in any case, the required values to calculate other sample sizes can be derived from the results presented in this study. In general terms, under the experimental conditions described here, the sample sizes (and consequently the reliability of ΔCPM) of all assessment methods tested are certainly acceptable for experimental or clinical use. It is important to note that this conclusion would otherwise not be reached if results were interpreted using arbitrarily fixed ICC or CV thresholds as the sole criteria for the evaluation of CPM reliability.

In particular, the NWR thresholds and the electrical pain detection thresholds display advantages over pain intensity ratings to suprathreshold electrical stimulation, since they present the highest rates of successful CPM induction (99% and 96%, respectively, versus 84%) and at the same time both measures require smaller sample sizes to detect the same effect. An additional advantage that the NWR thresholds hold compared to the other two methods is their objectivity, in the sense that the detection of the NWR threshold does not rely on a subjective assessment from the participant/patient or the experimenter/clinician [59]. On the other hand, electrical pain detection thresholds require the participants to inform at which intensity they start feeling pain (which is a subjective binary decision), and pain intensity ratings require further subjective evaluation (i.e., to come up with a value within a given scale that reflects the level of pain for a give stimulation intensity). However, the detection of NWR thresholds depends on the recording of EMG activity, something that it is not required in the other two methods. Ultimately, the decision of which measure to use will not solely depend on the reliability of these variables (which is acceptable in all cases) but on experimental or practical considerations, such us the level of objectivity needed or the type of equipment available.

## Conclusions

The interpretation of reliability indexes is not trivial, and should not be performed using fixed cut-off thresholds. Instead, well-defined clinical or analytical goals should be established in advance, and the assessment of reliability should be evaluated with regards to these goals, as for example sample sizes for potential future experiments. In relation to CPM assessment, it was demonstrated that under the experimental conditions presented in this study, the CPM paradigm is sufficiently reliable for experimental of clinical use. Moreover, the NWR threshold is recommended as test stimuli, since it is a strictly objective measure with a high level of reliability.

## Supporting Information

Protocol S1 **Study protocol.**
(PDF)

Checklist S1 **TREND statement checklist.**
(PDF)
