# Supplementary material for: Is the Conditioned Pain Modulation Paradigm Reliable? A Test-Retest Assessment Using the Nociceptive Withdrawal Reflex
Source: PLoS One. 2014 Jun 20;9(6):e100241. doi: 10.1371/journal.pone.0100241 (PMC4065000; doi:10.1371/journal.pone.0100241)
Supplement: Protocol S1 — Study protocol. (PDF) [file pone.0100241.s001.pdf]

Study Protocol

Version 5, 28.03.2012

Reliability of measuring conditioned pain modulation with the nociceptive withdrawal reflex

Pascal H. Vuilleumier*, MD

1st Author: Pascal H. Vuilleumier

Co-authors: Raphael Fritsche*, José A. Biurrun Manresa[@], Carmen Oehler*, Ole K. Andersen[@], Lars Arendt-Nielsen[@],

Senior author: Michele Curatolo*

*University Department of Anesthesiology and Pain Therapy, Inselspital, Bern, Switzerland.

[@]Center for Sensory-Motor Interaction, University of Aalborg, Denmark

Introduction

Background

Chronic pain is characterized by changes in the central processing of sensory inputs. Quantitative sensory tests (QST) explore central excitability in humans and have the potential to detect altered central pain processing in individual patients [1].

QST were developed to assess the responses to sensory stimuli for research purposes, providing psychophysical and electrophysiological methods for the assessment of the nociceptive system [1, 2]. Reliability is an essential condition for using QST in research and clinical practice. It can be defined as the consistency of measurements across time, patients or observers, and the extent to which it is error-free [3]. Three categories have been assessed for reliability in QST measures: intra-rater, inter-rater and test-retest reliability [4] [5]. Reliability of QST measures in healthy volunteers are encouraging in regard to good repeatability of QST measures over the time in the same subjects. Inter-rater reliability, a prerequisite to compare different measures in between different studies, are also highly encouraging [6] [7] [8] [9] [10].

Under normal conditions, pain after application of a test nociceptive stimulus is attenuated by the application of an additional "conditioning" noxious stimulus to a remote body region, reflecting diffuse endogenous inhibition [11, 12]. This is defined as conditioned pain modulation (CPM), also known as the "pain inhibits pain" paradigm. CPM has been object of much work in the last years. It goes back to the exploration of endogenous analgesia via descending pain-modulatory systems, which started about three decades ago in animal models [13]. Descending inhibitory pathways are under cerebral control, mediating modulation of pain perception by emotional, motivational and cognitive factors [13, 14]. Alterations of CPM are a known risk factor concerning acute and chronic pain syndromes [15].

Very few reliability studies on CPM have been conducted to date. To our knowledge, only S. Cathcart et al used occlusion cuff algometry and pressure pain detection to assess the test-retest reliability in healthy volunteers [16]. A study in chronic pain patients from our group has shown great variability in CPM (Schliessbach et al, unpublished.). It is unclear whether this phenomenon is related to high interindividual variability of the CPM measure itself, or to poor reliability. In an analysis of own data extracted from a pharmacological study, CPM measured at three different sessions in patients with low back pain displayed very poor reliability [17].

The nociceptive withdrawal reflex is an electrophysiological measure [18]. Compared to psychophysical measures which rely on the subject's pain perception, the reflex could be a more reproducible and stable measure over time [17].

Aims

The primary aim of the present study is to assess the reliability of CPM using the nociceptive withdrawal reflex as test stimulus and the ice water test as conditioning stimulus in 34 consecutive patients, with a 7 days interval between two sessions.

Secondary aim is to compare the reliability of CPM with the nociceptive withdrawal reflex to the reliability of CPM assessed with subjective pain assessments, i.e. pain threshold and pain intensity after electrical stimulation.

Methods

Design

Repeated assessments of CPM using the nociceptive withdrawal reflex threshold, electrical pain detection threshold and suprathreshold electrical stimulation of the skin, with ice-water as conditioning stimuli in all three models. There will be a 7 days interval between two measurements.

Setting

Pain Division of the Department of Anesthesiology and Pain Therapy, University Hospital, Inselspital Bern, Switzerland.

Subjects

We will test 34 consecutive pain-free volunteers. The volunteers will receive a compensation of 40 Swiss Francs for their participation.

Inclusion criteria:

- Male gender in order to eliminate cyclic hormonal variation.

Exclusion criteria:

- Age less than 18 or more than 65 years;
- Signs or suspicion of neurological disease or dysfunction;
- Ongoing treatment with any drug known to influence pain modulation: any analgesic, antidepressant, anticonvulsant or benzodiazepine;
- Intake of any analgesic drug during the 48h preceding the experiment.

Selection and recruitment

Subjects will be recruited in the Faculty of Medicine and the University Hospital of Bern with advertisement.

Pain tests

a- Mechanisms explored

The primary outcome variable will be the reliability of CPM by ice water test and nociceptive withdrawal reflex. The additional measures will be secondary outcomes. They are the reliability of CPM using the ice water test as conditioning stimulus for all models, the pain detection threshold and the pain rating to cutaneous electrical stimulation as test stimuli.

b- General methodological aspects

During the experimental session, patients will be positioned in a comfortable supine position, with the upper body elevated by 30°, in a closed and quiet room. The tests will be performed on the dominant body side.

Training sessions of the pain tests will be performed before starting the experiment, until the subjects will be familiar with the testing procedures.

In all volunteers the second pain test session will be performed at the same time of the day (±2 hours in regard to the first experimental session), in order to rule out possible circadian influences on pain sensitivity.

c- Electrical stimulation.

Electrical stimulation will be performed through bipolar surface Ag/AgCl-electrodes placed just distal to the lateral malleolus (innervation area of the sural nerve). Electromyographic (EMG) reflex responses to electrical stimulation will be recorded from the middle of the biceps femoris and the rectus femoris muscles (Ag/AgCl-electrodes). Stimulation and EMG recordings will be made by a computer-controlled constant current stimulator (NCS System, Evidence 3102 evo, Neurosoft, Russia).

A 25 ms, train-of-five, 1 ms, square-wave impulse (perceived as a single stimulus), will be delivered. The current intensity will be increased from 1 mA in steps of 1 mA until: 1) a biceps femoris reflex with an amplitude exceeding 20 μV for at least 10 ms in the 50-150 ms post-stimulation interval will be detected (single stimulus *reflex* threshold); and 2) a pain sensation will be evoked (single stimulus *pain* threshold). The single stimulus pain detection threshold will be multiplied by 1.5 to obtain a suprathreshold current intensity. Pain intensity after delivery of this suprathreshold stimulus will be assessed on the numerical rating scale (NRS), whereby 0=no pain and 10=worst pain imaginable.

d- Ice water test

The ice water test was used as conditioning stimulus. This test was chosen because it was shown to induce strong CPM in previous studies [19] [20].

The device consists of a container separated into an outer and an inner part by a mesh screen. The mesh screen prevents direct contact between the ice (placed in the outer part) and the hand of the subject (placed in the inner part). The water is regularly mixed to maintain the temperature in the inner part near to 0°C, as monitored by a thermometer with a digital display (±0.1°C).

The subject places his hand, wide open and to the wrist, into the inner part of the container. He is asked to keep it in the water until he feels an intolerable sensation of pain and is forced to remove his hand from the container, in any case for a maximum time of 2 min.

e- Conditioned pain modulation (CPM)

As explained in the introduction, this method explores the endogenous modulation of nociceptive input. Under normal conditions, pain after application of a test nociceptive stimulus is attenuated by the application of an additional "conditioning" noxious stimulus to a remote body region, reflecting diffuse endogenous inhibition [11, 12].

In the present study, the threshold of the nociceptive withdrawal reflex measured at the biceps femoris and the ice water test applied at the hand are used as primary "test" and "conditioning"

stimuli, respectively. An increase in the nociceptive withdrawal reflex threshold immediately after ice water test is an indication of efficient CPM.

The nociceptive withdrawal reflex threshold is measured again at the same time as the subject is withdrawing the hand from the water (one single measurement). CPM is measured as the difference in the thresholds between measurements after and before the ice water test.

The cutaneous electrical pain detection thresholds (secondary outcome) will be assessed at the same time as the threshold for the nociceptive withdrawal reflex. For the pain rating at suprathreshold stimulation (additional secondary outcome), the procedure with the ice water test will be repeated 30 min after the first recordings. This will be done in order to avoid a possible influence of the reflex measurement on a subsequent assessment of pain intensitiy.

Sample size considerations

The sample size calculation is based on two parameters: The detection of CPM and the reliability of the nociceptive withdrawal reflex.

a. Detection of CPM

CPM is measured as the difference in the stimulation threshold of the nociceptive withdrawal reflex between measurement after and before the ice water test. In a previous study, the nociceptive withdrawal reflex after single stimulation had a mean of 17 mA, a SD of 4 mA, and a range of 5-31 mA [21]. In order to detect a minimal difference of 2.0 mA between nociceptive withdrawal reflex threshold after and before ice water stimulation, with an expected SD of change of 4.0 mA, a probability of 80% that the study detects a difference at a two-sided significance level of 0.05, a paired t-test analysis yields a total of 34 patients.

b. Reliability

The sample size for a test-retest reliability study depends on the minimally acceptable level of reliability ρ_0, the target level of reliability ρ (measured as intraclass correlation coefficient), and the number of repetitions of the measurement n. The values for type I and type II errors (α and β) are set to 0.05 and 0.20, respectively [22]. Considering a minimum number of measurements of $n = 2$ (one measurement per session and two different sessions), reliability values of $\rho_0 = 0.5$ as the minimally accepted reliability and $\rho = 0.8$ as the expected reliability [10] [23], the minimum sample size for this experiment is 22 subjects.

In summary, 34 subjects are required in order to detect CPM and estimate test-retest reliability.

Statistical Analysis

The between-session reliability (also referred to as *stability over time*) of the nociceptive withdrawal reflex to single electrical stimulation will be assessed using multiple methods described below.

- *Intraclass correlation coefficient (ICC)*: it measures the relative homogeneity within sessions in relation to the total observed variation between sessions. For this analysis, a two-way mixed model using absolute agreement will be selected, and ICC for single measurements will be reported. ICC values above 0.75 are indicative of good reliability [24]. An *F* test with a hypothesized ICC value of 0.5

(representing a fair reliability level) will also be carried out in order to determine if the ICC values obtained are significantly different from this level.

- *Coefficient of variation (CV)*: it represents the standard error of measurement expressed as a percentage of the subject's average threshold. The CV can be interpreted as the percentage of deviation from the average threshold below which 68% of the differences between sessions may be expected to lie [25].

- *Bland-Altman agreement analysis*: it is based on the analysis of the average vs. the difference of the thresholds between two given sessions, from which the so called *limits of agreement (LA)* can be derived, as the average difference ± 1.96 times the standard deviation of the differences. The LA delimit the range within which 95% of the differences between thresholds in two single sessions may be expected to lie. In close relation to this definition, the *coefficient of repeatability (CR)* is defined as the value below which 95% of the *absolute* differences between thresholds in two single sessions may be expected to lie. If there is no systematic bias in the thresholds between sessions, the average difference between thresholds is expected to be zero, and the LA and the CR should be similar [26].

Relevance

To the knowledge of the authors, there has not been any study on the reliability of CPM with the nociceptive withdrawal reflex. Because this measurement is a reflex, and hence not dependent on subjective perception, more precise information and less variability in regard to spinal pain modulation is expected. Most studies on CPM have involved psychophysical QST measures, leaving a subjective error margin in the measured effects. If reliability of the CPM with the nociceptive withdrawal reflex can be established, this assessment could find increasing applications in clinical practice and research. By studying three different CPM measures, we will also be able to compare the reliability of electrophysiological and psychophysical CPM measures. Detection of individual disturbances in CPM may allow the development of mechanism-based interventions in pain therapy as well as a better understanding of the underlying physiopathology of chronic pain syndromes.

Varia

Risks

No risks related to the test procedures are known.

Ethical aspects

The pain measurements have been performed by our group for many years on several hundreds of subjects. They are well tolerated and currently used with children starting at age 7.

Data storage

The data will be stored in a locked-up drawer for 10 years at the University Hospital of Bern.

Financing and insurance

This study is part of a project financed by the Swiss National Science Foundation (SPUM no. 33CM30_124117) and the Scientific Funds of the University Department of Anesthesiology, Inselspital Bern, Switzerland.

The public liability of the investigators will be covered by the insurance of the Inselspital Bern.

Authorship

Authorship for the project will be based on the Uniform Requirements for Manuscripts of the International Committee of Medical Journal Editors (www.icmje.org/#author). According to these requirements, authorship credit should be based on 1) substantial contributions to conception and design, acquisition of data, or analysis and interpretation of data; 2) drafting the article or revising it critically for important intellectual content; and 3) final approval of the version to be published. Authors should meet conditions 1, 2, and 3.

Changes in the tasks of the authors listed below may lead to loss of authorship for some contributors or addition of new authors during the course of the project.

Tasks of Authors

Study design: all the authors.

Draft of the study protocol: Pascal Vuilleumier, José A. Biurrun Manresa and Michele Curatolo

Logistic organisation: Pascal Vuilleumier, Raphael Fritsche and Carmen Oehler.

Performance of the experiments: Pascal Vuilleumier and Raphael Fritsche.

Analysis of data of pain tests: José A. Biurrun Manresa.

Interpretation of findings: all authors.

Draft of article: Pascal Vuilleumier and Raphael Fritsche, assisted by Michele Curatolo.

Final version of article: all authors.

Senior responsibility of all processes: Michele Curatolo.

The study will be the dissertation of Raphael Fritsche.

Time plan

Patients recruitment: from Mai 2012 to September 2012

Data analysis: November 2012.

First draft of paper: January 2013.

Submission to peer-reviewed journal: March 2013.

References

1. Curatolo, M., *Diagnosis of altered central pain processing.* Spine (Phila Pa 1976), 2011. **36**(25 Suppl): p. S200-4.
2. Arendt-Nielsen, L. and D. Yarnitsky, *Experimental and clinical applications of quantitative sensory testing applied to skin, muscles and viscera.* J Pain, 2009. **10**(6): p. 556-72.
3. Portney, L.G., Watkins, M.P., *Foundations of clinical research.* Applications to practice, 2000. **2nd edition**(Upper Saddle River, NJ: Prentice Hall Health).
4. Chong, P.S. and D.P. Cros, *Technology literature review: quantitative sensory testing.* Muscle Nerve, 2004. **29**(5): p. 734-47.
5. Moloney, N.A., et al., *Reliability of thermal quantitative sensory testing of the hand in a cohort of young, healthy adults.* Muscle Nerve, 2011. **44**(4): p. 547-52.
6. Wasner, G.L. and J.A. Brock, *Determinants of thermal pain thresholds in normal subjects.* Clin Neurophysiol, 2008. **119**(10): p. 2389-95.
7. Agostinho, C.M., et al., *Habituation and short-term repeatability of thermal testing in healthy human subjects and patients with chronic non-neuropathic pain.* Eur J Pain, 2009. **13**(8): p. 779-85.
8. Chesterton, L.S., et al., *Interrater reliability of algometry in measuring pressure pain thresholds in healthy humans, using multiple raters.* Clin J Pain, 2007. **23**(9): p. 760-6.
9. Jones, D.H., R.D. Kilgour, and A.S. Comtois, *Test-retest reliability of pressure pain threshold measurements of the upper limb and torso in young healthy women.* J Pain, 2007. **8**(8): p. 650-6.
10. Micalos, P.S., et al., *Reliability of the nociceptive flexor reflex (RIII) threshold and association with Pain threshold.* Eur J Appl Physiol, 2009. **105**(1): p. 55-62.
11. Chitour, D., A.H. Dickenson, and D. Le Bars, *Pharmacological evidence for the involvement of serotonergic mechanisms in diffuse noxious inhibitory controls (DNIC).* Brain Res, 1982. **236**(2): p. 329-37.
12. Ge, H.Y., P. Madeleine, and L. Arendt-Nielsen, *Sex differences in temporal characteristics of descending inhibitory control: an evaluation using repeated bilateral experimental induction of muscle pain.* Pain, 2004. **110**(1-2): p. 72-8.
13. Pud, D., Y. Granovsky, and D. Yarnitsky, *The methodology of experimentally induced diffuse noxious inhibitory control (DNIC)-like effect in humans.* Pain, 2009. **144**(1–2): p. 16-19.
14. Gebhart, G.F., *Descending modulation of pain.* Neurosci Biobehav Rev, 2004. **27**(8): p. 729-37.
15. Yarnitsky, D., *Conditioned pain modulation (the diffuse noxious inhibitory control-like effect): its relevance for acute and chronic pain states.* Curr Opin Anaesthesiol, 2010. **23**(5): p. 611-5.
16. Cathcart, S., et al., *Reliability of temporal summation and diffuse noxious inhibitory control.* Pain Res Manag, 2009. **14**(6): p. 433-8.
17. Neziri, A.Y., et al., *Effect of intravenous tropisetron on modulation of pain and central hypersensitivity in chronic low back pain patients.* Pain, 2012. **153**(2): p. 311-8.
18. Geber, C., et al., *Comparison of LEP and QST and their contribution to standard sensory diagnostic assessment of spinal lesions: a pilot study.* Neurol Sci, 2011. **32**(3): p. 401-10.
19. Willer, J.C., A. Roby, and D. Le Bars, *Psychophysical and electrophysiological approaches to the pain-relieving effects of heterotopic nociceptive stimuli.* Brain, 1984. **107 (Pt 4)**: p. 1095-112.
20. Willer, J.C., T. De Broucker, and D. Le Bars, *Encoding of nociceptive thermal stimuli by diffuse noxious inhibitory controls in humans.* J Neurophysiol, 1989. **62**(5): p. 1028-38.
21. Neziri, A.Y., et al., *The nociceptive withdrawal reflex: normative values of thresholds and reflex receptive fields.* Eur J Pain, 2010. **14**(2): p. 134-41.
22. Walter, S.D., M. Eliasziw, and A. Donner, *Sample size and optimal designs for reliability studies.* Stat Med, 1998. **17**(1): p. 101-10.

23. Rhudy, J.L. and C.R. France, *Defining the nociceptive flexion reflex (NFR) threshold in human participants: a comparison of different scoring criteria.* Pain, 2007. **128**(3): p. 244-53.
24. Portney, L., Watkins MP, *Foundations of clinical research: applications to practice.* Pearson/Prentice Hall, Upper Saddle River, NJ, 2009.
25. Atkinson, G. and A.M. Nevill, *Statistical methods for assessing measurement error (reliability) in variables relevant to sports medicine.* Sports Med, 1998. **26**(4): p. 217-38.
26. Bland, J.M. and D.G. Altman, *Measuring agreement in method comparison studies.* Stat Methods Med Res, 1999. **8**(2): p. 135-60.

Pascal H. Vuilleumier, 28.03.2012
